# Factors associated with non-adherence to antiretroviral therapy among adolescents in Oshikoto Region, Namibia

**DOI:** 10.4102/safp.v67i1.6020

**Published:** 2025-02-28

**Authors:** Daniel O. Ashipala, Tukwatha A. Shuukwanyama, Abraham V. Nghikevali

**Affiliations:** 1 Department of General Nursing Sciences, Faculty of Health Sciences and Veterinary Medicine, University of Namibia, Rundu, Namibia

**Keywords:** non-adherence, antiretroviral, therapy, adolescents, Namibia

## Abstract

**Background:**

In Namibia, the Omuthiya Antiretroviral Therapy (ART) Clinic has indicated that nine older adolescents (15–19 years) out of 125 adolescents active on ART have defaulted and become lost to care. This represents an 89% retention rate among older adolescents compared to a 97% retention rate for the adult population in the same facility. There is a lack of information on the extent of ART adherence among older adolescents, particularly in terms of viral suppression rates. This study aims to measure the level of adherence to ART treatment among adolescents enrolled at the Omuthiya ART Clinic in the Oshikoto Region, Namibia.

**Methods:**

From November to December 2020, a cross-sectional study was conducted among 100 adolescents living with HIV (ALHIV) at the Omuthiya ART Clinic. Data were collected through structured questionnaires and analysed using descriptive statistics and logistic regression.

**Results:**

The study found that 24% of adolescent patients on ART treated at the Omuthiya ART Clinic are non-adherent to treatment. A significant association found was between education level and adherence (*p* = 0.008), alcohol use and adherence (*p* = 0.022) and memory aids use and adherence (*p* < 0.001).

**Conclusion:**

Adolescents’ adherence to ART at the Omuthiya ART Clinic is influenced by educational level, alcohol use, and memory aid use. Further research is needed to explore other potential factors influencing adherence.

**Contribution:**

These findings may be used to develop ongoing strategies and targeted interventions that prioritise a patient-centred care approach, recognising the autonomy of adolescents. In addition, they highlight the importance of a supportive family environment in managing HIV and improving ART adherence.

## Introduction

Children and adolescents account for approximately 10% of the 36.9 million people living with HIV worldwide, and about 80% come from sub-Saharan Africa.^1^ Global paediatric ART coverage has increased from 44% in 2014 to 52% in 2017, but still falls short of the Start Free, Stay Free, AIDS Free framework of reaching 95% of children and adolescents living with HIV (ALHIV) with lifesaving antiretroviral therapy (ART).^2^ Namibia is one of the 21 priority countries under the UNAIDS Start Free, Stay Free, AIDS Free framework, with targets to end AIDS among children, adolescents and young women and reach 95% treatment coverage by 2020.^3^ According to the study carried out by the Ministry of Health and Social Services (MoHSS)^4^ in Namibia, there are approximately 185 000 people living with HIV, of whom 9000 are children between 0 and 14 years and 4955 are adolescents between 15 and 19 years.^3^ The MoHSS^5^ further reported that improved access to HIV treatment and care for children has resulted in greater numbers of HIV-positive infants surviving into adolescence.^3^ The viral load suppression among young people aged 15–24 living with HIV is lower at 65.4% compared to the adult viral load suppression of 92.5%.^3^ This age group includes individuals who acquired HIV both perinatally and through other means, such as sexual transmission. Adolescents infected perinatally often face unique challenges, such as complex treatment histories, developmental issues and psychosocial factors associated with managing a lifelong illness.^6,7^ On the other hand, those who acquired HIV during adolescence may encounter different barriers, including stigma, a lack of disclosure and unfamiliarity with healthcare systems.^8^ Tailoring adherence support to address these distinct challenges can help improve viral load suppression outcomes among young people living with HIV.

The MoHSS ART guideline indicates that viral suppression is higher among the 10–14-year-olds at 73% compared to 63% among the 15–19-year-olds.^5^ Data from the MoHSS^4^ show that viral load suppression among young people aged 15–24 years living with HIV is lower at 65.4% for females compared to 86% for males.^2^ The National ART Guideline further suggests that older adolescents (15–19 years) adhere less to the medication than younger children. Evidence suggests that among those aged 15–24, many are individuals who have progressed from childhood HIV rather than newly diagnosed cases. These perinatally infected adolescents face unique adherence challenges, which can impact their understanding of the importance of adherence.^9,10^ Newly diagnosed youth struggle with stigma and mental health issues, impacting their adherence to ART.^11^ Recognising these distinctions is essential for tailoring adherence support to the specific needs of these subgroups.

Adherence to ART is even more complicated because it is very time sensitive.^2^ Adherence to ART is critical for maintaining long-term virological suppression, and ultimately the quality of life. Maintaining high levels of adherence is a daily challenge and a dynamic process in the life of an adolescent living with HIV.^2^ Adolescents face many challenges related to their age and the different biological, physiological, social and behavioural changes that they go through. This study has significant scientific and social value, as it addresses the pressing issue of ART adherence among adolescents, a group with notably lower viral suppression rates compared to adults. From a scientific perspective, understanding the specific adherence challenges faced by adolescents can inform targeted interventions that improve ART adherence and viral load suppression. This study also contributes to the knowledge base on HIV care in a sub-Saharan African context, where HIV prevalence is high, and where unique cultural and systemic factors impact adherence.

Socially, improving adherence among adolescents is vital for reducing HIV transmission within communities, enhancing the quality of life for young people living with HIV, and helping Namibia progress towards the UNAIDS 95-95-95 goals. The study unpacks the broader impact of non-adherence, highlighting the risk of drug resistance, continued transmission and psychosocial stress on affected adolescents. Ensuring that young people remain virally suppressed helps reduce stigma associated with HIV.^12^ This study seeks to address the existing gap in knowledge concerning the specific adherence challenges faced by adolescents who have lived with HIV since childhood compared to those who are newly diagnosed during adolescence. Specifically, it aims to measure the level of adherence to ART among adolescents enrolled at the Omuthiya ART Clinic in the Oshikoto Region of Namibia, and to propose recommendations based on the findings.

## Methods

### Study design and setting

A descriptive cross-sectional survey study using a structured questionnaire was applied to obtain data from adolescents enrolled on ART at the Omuthiya ART Clinic, Omuthiya District in the Oshikoto region. This design is relatively inexpensive and can be used within a short period.^13^ Ten facilities in the Omuthiya district offer ART services to ALHIV. These include Omuthiya ART Clinic, Omuthiya PHC Clinic, Amilema Clinic, Onanke Clinic, Onyuulaye Clinic, Onamishu Clinic, Onkumbula Clinic and Hedimbi Clinic. The Omuthiya ART clinic is the busiest site with 262 patients, of which 125 were adolescents in its care.^14^ Adolescents in this study attend the same clinic. However, membership in the Teen Club is not mandatory.

### Study population, sampling and inclusion criteria

The target population for this study was all adolescents with HIV in the Omuthiya district, that is 125 patients. The study aimed to focus specifically on those attending the Omuthiya ART clinic, as this clinic serves as a vital and only resource for HIV-positive adolescents. In this study, the population and the sample were the same because of the small size of the target population; hence, the researchers included all individuals. Out of the total population, only 100 adolescents from the targeted 125 participated in the study. There was no sampling method applied in this study.

### Data collection and statistical analysis

Data were collected in December 2020 using a pre-tested questionnaire during participants’ ART follow-up at the facility. Prior to the actual study, the data collection instrument underwent a pilot test with six adolescents who were not included in the main research. The pilot study was conducted in this study with the aim to assess the feasibility and clarity of the research instrument, ensuring that the questions were relevant and easily understood by the target population. Following the pilot, feedback was analysed to identify any areas for improvement. This process included assessing item clarity and relevance, as well as conducting reliability tests to measure the consistency of responses. Adjustments were made based on participant feedback, and the final version of the questionnaire was validated to ensure it effectively captured the necessary data for the study. This thorough validation process not only enhanced the instrument’s reliability but also ensured it was attuned to the unique experiences and needs of ALHIV.

The data were entered into Statistical Package for Social Sciences (SPSS) version 26 for analysis. To ensure the quality of the data entered, double-entry was performed to address discrepancies and the data were cleaned before the analysis. Descriptive statistics in the form of frequencies and percentages were used. Cross-tabulations were conducted using the Chi-square test to assess associations between categorical variables. Binary logistic regression was performed to identify predictors of adherence, with a *p*-value of less than 0.05 considered statistically significant at a 95% confidence interval. Adherence was assessed based on pill counts, with good adherence defined as missing less than two daily doses per month.

### Ethical considerations

The research was conducted after approval was granted by the School of Nursing Research and Ethics Committee of the Faculty of Health Sciences, University of Namibia, Namibia (reference no.: SoNREC 44/2020). Permission to conduct the study was also sought from the Ministry of Health and Social Services, reference number 17/3/3TAS. Informed consent was sought verbally from the participating participants, and through their signing of a consent form. Anonymity and confidentiality were ensured. Confidentiality was ensured by the use of pseudonyms on the research tools instead of writing names.

## Results

This study targeted adolescents LWHIV. Initially, 125 adolescents aged 15–19 years were identified as those who met the inclusion criteria for the study. However, upon further review, only 100 adolescents actually participated. This exclusion was primarily because of two factors: some adolescents did not meet the age criteria, which included being aged 15–19 years, while others chose not to participate in the study. The study selection process was not intended to randomly exclude individuals; rather, it reflected the specific requirements set for the study, as well as the realities of participation. Ultimately, these 100 adolescents were selected as the study sample, to provide experiences of HIV-positive youth within this community.

Descriptive statistics were conducted to profile the study participants, revealing several important and statistically significant findings (see Table 1). The majority of participants (60%) were female, with a substantial proportion (73%) having completed secondary education. Notably, 81% of participants exhibited suppressed or low-level viral loads, indicating effective disease management, although 19% with high viral loads highlight a need for targeted intervention. Medication adherence varied, with 30% of those on daily doses demonstrating good adherence and 15% showing potential issues, while 9% of those on twice-daily doses exhibited significant non-adherence.

**TABLE 1:** Frequency distribution of participants’ socioeconomic and demographic characteristics (*N* = 100).

Characteristic	Number of participants (*n*)	%
**Socioeconomic and demographic characteristics of the participants**		
Sex:		
Male	40	40
Female	60	60
Education level:		
Primary	21	21
Secondary	73	73
Tertiary	6	6
**Medical history**		
Current viral load status:		
Virally suppressed (0–40)	47	47
Low level viraemia (41–999)	34	34
High viral load (> 1000)	19	19
Current regimen classification:		
1st line	60	60
2nd line	39	39
3rd line	1	1
Are you on dolutegravir (DTG) based regimen?		
Yes	89	89
No	11	11
On average how, many pills remain during your clinical visit?		
Daily doses (1 tablet per day):		
< 2	30	30
2–4	28	28
> 5	15	15
Bis in die (BD) doses (2 tablets per day):		
< 3	7	7
4–8	11	11
> 9	9	9
**Personal history**		
Are you staying with your own parents?		
Yes	64	64
No	36	36
Do you keep your medication yourself?		
Yes	89	89
No	11	11
How many meals do you have per day?		
One	33	33
Two	46	46
Three or more	21	21
**Adherence**		
How often do you forget to take your ARV?		
Sometimes (forget more than 3 doses in a month)	64	64
Always	12	12
Rarely	24	24
Are you using alcohol to the extent that it makes you forget to take your ARV?		
Never	94	94
Sometimes	6	6
Do you experience lack of sleep or stress due to your HIV status?		
Yes	14	14
No	86	86
**Health centre assistance**		
During your follow-up visit at a clinic, were you provided with sufficient information on the importance of taking antiretroviral (ARV) medication every day and correctly?		
Sometimes	41	41
Always	54	54
Rarely	5	5
Is there a time when you came to a clinic and you were sent back home without medication because there was no medication or nurses or you missed your follow up date?		
Yes	7	7
No	93	93
How long do you stay at a clinic during your visit?		
< 1 h	28	28
1 h – 2 h	42	42
> 2 h	30	30
Was there a time when you missed your follow-up date because you did not have transport money to come a clinic?		
Yes	33	33
No	67	67
**Taking medication**		
How many pills do you take per day?		
One	73	73
Two to four	25	25
More than four	2	2
Have you ever experienced an adverse reaction after taking your medication for example rashes, vomiting, tiredness et cetera?		
Sometimes	52	52
Always	6	6
Rarely	42	42
Do you use memory aids such as a timer, alarm clock or cell phone to remind you to take your medicine?		
Sometimes	31	31
Always	45	45
Rarely	24	24
How often does your caregiver remind you to take your medication?		
Everyday	48	48
Some days	29	29
Never	22	22
**HIV and/or AIDS Stigma Experience**		
Have you disclosed your HIV status to any of your peers at school?		
Yes	17	17
No	83	83
Do you take your pill bottles with you to school?		
Yes	15	15
No	85	85
Have you ever experienced any form of stigmatisation at school, home or in the community based on your HIV status?		
Sometimes	44	44
Always	5	5
Rarely	51	51
Are you a member of a teen club for adolescents living with HIV at your clinic?		
Yes	54	54
No	46	46
**Cultural Beliefs**		
Have you ever stopped your ARV medication because of any cultural or religious beliefs?		
Yes	5	5
No	95	95
Do you mix your ARV medication with traditional herbs?		
Yes	4	4
No	96	96

Patient-related barriers were notable; 87% managed their own medications, yet only 24% reported rarely forgetting to take their ARVs (see Table 1). Economic constraints were evident, as 46% had only two meals per day, potentially impacting overall health and adherence. Social support factors indicated that 64% lived with their parents, providing some potential assistance, but 52% felt that ARVs rarely affected their school performance. Despite potential stigma, 94% reported not using alcohol to an extent that interfered with their adherence. Support from health centres was adequate, with 54% receiving sufficient adherence information, and 93% never sent home without their medication. Most participants (73%) took only one pill per day, with 52% occasionally facing adverse reactions to their medication. In terms of adherence reminders, 45% utilised electronic reminders, and 48% relied on caregivers. Disclosure of HIV status to peers was low, with only 17% sharing this at school. Importantly, 96% did not mix ARVs with traditional herbs, indicating a commitment to adhering to their prescribed treatments.

The results in Table 2 present the *p*-values from a chi-square test examining the association between various explanatory variables and adherence to ART among adolescents at the Omuthiya ART Clinic. With the alpha level at 0.05, there is a significant association (*p* = 0.008) between the education level of adolescents and their adherence to ART, indicating that higher education levels positively impact adherence. Alcohol use has a significant impact on ART adherence (*p* = 0.022), suggesting that adolescents who use alcohol to the extent that it affects their ability to take ARVs are less likely to adhere to their treatment. The use of memory aids such as timers, alarm clocks or cell phones to remind adolescents to take their medication shows a significant association with adherence (*p* < 0.001). This indicates that memory aids are effective in improving ART adherence among adolescents. No significant associations were found between other personal characteristics, medical history, personal history, health centre assistance, stigma experiences or cultural beliefs and ART adherence in this study.

**TABLE 2:** Cross tabulation of explanatory variables and adherence to antiretroviral therapy among adolescents.

Adherence	Non-adherence	Adherence	Total (*f* [%])	χ^2^	*df*	*p*
Personal characteristics * adherence						
Sex:	-	-	-	1.515	1	0.218
Male	15	-	40	-	-	-
Female	30	-	60	-	-	-
Total	45	55	100	-	-	-
Education level:	-	-	-	10.975	2	0.004*
Primary	15	6	21	-	-	-
Secondary	25	47	72	-	-	-
Tertiary	5	2	7	-	-	-
Total	45	55	100	-	-	-
Religion:	-	-	-	5.130	3	0.163
ELCIN	24	36	60	-	-	-
Anglican	9	12	21	-	-	-
Catholic	9	7	16	-	-	-
Other	3	0	3	-	-	-
Total	45	55	100	-	-	-
Medical history * adherence						
Current viral load status:	-	-	-	1.714	2	0.424
Virally suppressed (0–40)	21	23	44	-	-	-
Low level viraemia (41–999)	18	19	37	-	-	-
High viral load (> 1000)	6	13	19	-	-	-
Total	45	55	100	-	-	-
Current regimen classification:	-	-	-	1.336	2	0.513
1st line	29	31	60	-	-	-
2nd line	16	23	39	-	-	-
3rd line	0	1	1	-	-	-
Total	45	55	100	-	-	-
Are you on dolutegravir (DTG)-based regimen?	-	-	-	0.372	1	0.750
Yes	41	48	89	-	-	-
No	4	7	11	-	-	-
Total	45	55	100	-	-	-
On average, how many pills remain during your clinical visit?	-	-	-	3.303	3	0.347
Daily doses (1 tablet per day):	-	-	-	-	-	-
< 2	21	16	37	-	-	-
2–4	9	14	23	-	-	-
> 9	5	8	13	-	-	-
Not applicable	10	17	27	-	-	-
Total	45	55	100	-	-	-
Bis in die (BD) doses (2 tablets per day):	-	-	-	3.727	3	0.292
< 3	1	7	8	-	-	-
4–8	7	8	15	-	-	-
> 9	2	2	4	-	-	-
Not applicable	35	38	73	-	-	-
Total	45	55	100	-	-	-
Personal history * adherence						
Are you staying with your own parents?	-	-	-	1.217	1	0.270
Yes	31	32	64	-	-	-
No	14	23	36	-	-	-
Total	45	55	100	-	-	-
Do you keep your medication yourself?	-	-	-	2.777	1	0.249
Yes	40	49	89	-	-	-
No	4	7	11	-	-	-
Total	44	56	100	-	-	-
How many meals do you have per day?	-	-	-	0.104	2	0.949
One	15	18	33	-	-	-
Two	20	26	46	-	-	-
Three or more	10	11	21	-	-	-
Total	45	55	100	-	-	-
Adherence factors * adherence						
How often do you forget to take your ARV?	-	-	-	4.545	2	0.103
Sometimes (forget more than 3 doses in a month)	24	40	64	-	-	-
Always	6	6	12	-	-	-
Rarely	15	9	24	-	-	-
Total	45	55	100	-	-	-
Are you using alcohol to the extent that it makes you forget to take your ARV?	-	-	-	5.222	1	0.022*
Never	45	49	94	-	-	-
Sometimes	0	6	6	-	-	-
Total	45	55	100	-	-	-
Do you experience a lack of sleep or stress because of your HIV status?	-	-	-	1.343	2	0.511
Yes	5	9	14	-	-	-
No	40	46	86	-	-	-
Total	45	55	100	-	-	-
Health centre assistance * adherence						
During your follow-up visit at a clinic, were you provided with sufficient information on the importance of taking antiretroviral (ARV) medication every day and correctly?	-	-	-	2.248	2	0.325
Sometimes	15	26	41	-	-	-
Always	28	26	54	-	-	-
Rarely	2	3	5	-	-	-
Total	45	55	100	-	-	-
Is there a time when you came to a clinic and you were sent back home without medication because there was no medication or nurses or you missed your follow up date?	-	-	-	1.698	2	0.428
Yes	2	5	7	-	-	-
No	43	50	93	-	-	-
Total	45	55	100	-	-	-
How long do you stay at a clinic during your visit?	-	-	-	0.448	2	0.746
Less than 1 h	14	14	28	-	-	-
1 to 2 h	19	24	43	-	-	-
More than 2 h	12	17	29	-	-	-
Total	45	55	100	-	-	-
Was there a time when you missed your follow-up date because you did not have transport money to come a clinic?	-	-	-	0.377	2	0.828
Yes	14	19	33	-	-	-
No	31	36	67	-	-	-
Total	45	55	100	-	-	-
Taking medication * adherence						
How many pills do you take per day?	-	-	-	3.505	2	0.223
One	33	32	65	-	-	-
Two to four	12	21	33	-	-	-
More than four	0	2	2	-	-	-
Total	45	55	100	-	-	-
Have you ever experienced an adverse reaction after taking your medication for example rashes, vomiting, tiredness etc.?	-	-	-	2.0971	2	0.352
Sometimes	24	28	52	-	-	-
Always	1	5	6	-	-	-
Rarely	20	22	42	-	-	-
Total	45	55	100	-	-	-
Do you use memory aids such as a timer, alarm clock or cell phone to remind you to take your medicine?	-	-	-	100.000	2	< 0.001*
Sometimes	0	31	31	-	-	-
Always	45	0	45	-	-	-
Rarely	0	24	24	-		-
Total	45	55	100	-	-	-
How often does your caregiver remind you to take your medication?	-	-	-	3.336	3	0.343
Everyday	25	23	48	-	-	-
Some days	13	16	29	-	-	-
Never	7	16	23	-	-	-
Total	45	55	100	-	-	-
HIV and/or AIDS Stigma Experience * adherence						
Have you disclosed your HIV status to any of your peers at school?	-	-	-	10.728	1	0.121
Yes	7	10	17	-	-	-
No	38	45	83	-	-	-
Total	45	55	100	-	-	-
Do you take your pill bottles with you to school?	-	-	-	1.737	2	0.420
Yes	6	9	15	-	-	-
No	39	46	85	-	-	-
Total	45	55	100	-	-	-
Have you ever experienced any form of stigmatisation at school, home or in the community based on your HIV status?	-	-	-	2.518	2	0.284
Sometimes	16	28	44	-	-	-
Always	3	2	5	-	-	-
Rarely	26	25	51	-	-	-
Total	45	55	100	-	-	-
Are you a member of a teen club for adolescents living with HIV at your clinic?	-	-	-	2.227	1	0.136
Yes	28	26	54	-	-	-
No	17	29	46	-	-	-
Total	45	55	100	-	-	-
Cultural Beliefs * adherence						
Have you ever stopped your ARV medication because of any cultural or religious beliefs?	-	-	-	0.053	1	0.818
Yes	2	3	5	-	-	-
No	43	52	95	-	-	-
Total	45	55	100	-	-	-
Do you mix your ARV medication with traditional herbs?	-	-	-	-	-	-
No	45	55	100	-	-	-
Total	45	55	100	-	-	-

* , *p*-value less than 0.05.

Data analysis to test which factors affect adherence was conducted using the binary logistic regression method because there were two outcomes (adherence and non-adherence). Table 3 shows the results of the analysis. The results from the binary logistic regression analysis reveal several key points about the model’s performance and the significance of variables. The model accurately predicted adherence 89.1% of the time and non-adherence 33.3% of the time, leading to an overall correct classification rate of 64%. This indicates that while the model performs reasonably well in identifying adherent individuals, it struggles to predict non-adherence effectively.

**TABLE 3:** Binary logistic regression.

Observed	Variable	Predicted			Variables in the equation						95% CI for Exp (*β*)		
		Adherence		Percentage correct	*β*	s.e.	Wald	*df*	Sig.	Exp (*β*)	Lower	Upper
		Non-adherence	Adherence									
Step 1	Non-adherence	15	30	33.3	-	-	-	-	-	-	-	-
	Adherence	6	49	89.1	-	-	-	-	-	-	-	-
	Overall Percentage	-	-	64.0	-	-	-	-	-	-	-	-
	Constant	-	-	-	0.201	0.201	0.997	1	0.318	1.222	-	-
	Education level	-	-	-	0.550	0.410	1.802	1	0.001	1.733	0.776	3.870
	Memory aids	-	-	-	−0.186	0.278	0.448	1	< 0.001	0.830	0.481	1.431

*β*, beta; s.e., standard error; *df*, degrees of freedom; Sig., significance; Exp *β*, exponential of beta; CI, confidence interval.

† , The cut-off value is 0.500.

The constant term was not statistically significant (*p* = 0.318), meaning that it does not significantly differ from zero in predicting the outcome. Examining the specific variables, the coefficient for education level is positive (0.550), suggesting a trend towards increased odds of adherence with higher education levels. This effect is statistically significant (*p* = 0.001), indicating that education level has a reliable impact on adherence in this model. Similarly, the variable regarding the use of memory aids has a negative coefficient (-0.186), implying that using memory aids might be associated with lower adherence odds in those already non-adherents. This result is also statistically significant (*p* ≤ 0.001). Most other variables, including gender, religion, and various aspects of adherence behaviour (e.g. frequency of ARV taking, stigmatisation experiences), were not statistically significant in the binary logistic regression model at 95% level of significance.

## Discussion

This study aimed to identify factors associated with non-adherence to ART among adolescents at the Omuthiya ART Clinic in the Oshikoto Region of Namibia. The results highlight several key demographics, personal, and medical characteristics of the participants and their adherence behaviours. According to the national ART guideline, less than two daily dose pills count are indication of good adherence, 2–4 daily doses pills count portray fair adherence, and more than five was an indication of poor adherence. The study revealed that, most of the adolescents had notable suppressed or low viral loads, indicating effective management of their HIV. However, 19% had high viral loads and this did not include those that had defaulted from attending the clinic, necessitating targeted interventions. The majority of patients were on a first-line regimen, with fewer on second-line and third-line treatments, reflecting the typical progression of HIV treatment. A high percentage were on a DTG-based regimen, aligning with current treatment guidelines favouring DTG for its efficacy and tolerability. Viral load monitoring is an objective measurement of adherence in people on ART.

Our study found that most of the adolescents have two or more meals per day. Food availability and adherence to ART were found to be independent variables in this study. This was supported by the study performed by Nagata et al.^15^ Strong plausible evidence of the association between non-adherence to ART and food insecurity was found. Food insecure PLWHA were found to be twice as likely to display non-adherence to ART than their food secure counterparts. Studies in both developing and developed countries have indicated a strong association between non-adherence to ART and food security effects. For example, studies in Lake Victoria, Kenya^15^ and in Atlanta, USA showed that food insecure PLWHA miss doses of their daily medication.^16^ Similarly, studies in Jimma, southwest Ethiopia,^17^ and Congo^18^ established a strong association between non-adherence to ART and food insecurity. Because non-adherence to ART can risk developing viral resistance,^18,19^ food insecurity poses a significant challenge to the HIV and/or AIDS response.^20,21,22^

Chi-square tests revealed no significant association between most personal characteristics and adherence, except for education level (*p* = 0.008). Lack of sleep and stress because of HIV status were significantly associated with non-adherence (*p* = 0.022). Memory aids (timers, alarms, cell phones) were significantly associated with better adherence (*p* < 0.001). Stigma and cultural beliefs were not significantly associated with adherence. The findings align with several studies on adolescent ART adherence. For example, MacCarthy et al.^23^ highlighted the role of psychological support in improving ART adherence among adolescents. Similarly, a study by Slogrove et al.^24^ found that higher education levels correlate with better adherence, supporting the significant association found in this study.

However, some studies present conflicting findings. A study by Vreeman et al.^25^ reported a stronger impact of stigma on ART adherence than observed in this study, suggesting that regional or cultural differences may influence the extent to which stigma affects adherence. In addition, while this study found no significant impact of cultural beliefs on adherence, other research, such as that by Hlophe et al.,^26^ has shown that cultural beliefs can significantly hinder ART adherence in some communities.

The use of memory aids was significantly associated with better adherence in this study, which is consistent with findings by Chhim et al.,^27^ who reported that electronic reminders significantly improve medication adherence among adolescents. This is similar to a study conducted by Gross et al.,^28^ which showed that patients who use reminders to take their medications were more likely to have better adherence than those who did not use any reminders.^28^ Patients who use memory aids were three times more likely to adhere to treatment than those who did not use any memory aids.^29^ Among the adherent group in this study, those that use reminders to take treatment (91.7%) were more adherent than those that did not use any reminders (85.3%). This suggests that patients should be encouraged to use reminders regularly to ensure that they take medication as prescribed. However, among the non-adherent group, those that used the reminders to take treatment (8.3%), adhered less compared to those that did not use any reminder (14.7%). This suggests that other factors other than having reminders among the non-adherents are involved.^29^

The model’s overall classification performance is moderate (64% accuracy), but it shows a strong ability to correctly identify adherent cases while failing to correctly classify non-adherent cases. The analysis suggests that education level and the use of memory aids are significant predictors of adherence. Specifically, those who use memory aids are more likely to adhere to their medication regimen. Many variables, including gender, religion, and various experiences related to stigma or treatment side effects, do not show significant relationships with adherence in this model. The results inform that there is a need to consider refining the model by including additional predictors or interactions between variables to improve the model’s ability to classify non-adherence. Explore significant predictors such as a focus on understanding how memory aids influence adherence to develop targeted interventions.

## Strength

The results of the study may help the MOHSS to understand the challenges facing adolescents on ART and to tackle them in order to improve health of ALHIV. The inferences made from these data can be used to develop interventions that are relevant to the specific contexts and environments in which the patients live on a daily basis. When further developed and analysed from the patient’s perspective they can be used to inform policymakers on how quality of care can be improved and better understand the drivers of social determinants of health affecting young people.

## Limitations

This study was conducted at one local ART site. This limits the generalisation of the research findings to the rest of the ART clinics in Namibia. The reliance on convenience sampling may result in bias, as those participants choosing not to participate may well include patients with poor adherence. Furthermore, the study’s dependence on self-reported data via questionnaires could introduce social desirability bias, causing participants to respond in ways they believe are socially acceptable. The sample of the study was relatively small to represent all the ART adolescents in the country. Thus, in order to generalise the results from this study to other contexts, a bigger study needs to be conducted with the incorporation of other clinics in different regions of Namibia. Furthermore, the research instrument contained closed-ended questions, which meant it limited the response options for the participants. The fact that the model performs well for predicting adherence but poorly for non-adherence indicates that there might be additional factors influencing non-adherence that are not captured by the current model.

Another limitation was the lack of granular information on length of time on ART for new or perinatal exposure and the age of disclosure by care givers. These may have provided more information on factors affecting adherence. The research instrument also had a limitation in that the questionnaire contained only closed and open-ended questions, which meant limited response options, by implication. The research investigation was conducted in only one district, Oshikoto, the population and sample have limited statistic value and therefore results could not be generalised. To generalise the findings a bigger study should be conducted.

## Conclusion

This study concludes that 24% of adolescent patients on ARV treated at the Omuthiya ART Clinic are non-adherent to treatment. It was concluded that there is no significant association between the personal characteristics of participants and adherence to ART. A significant association was found between education level and adherence; alcohol consumption and adherence; and the use of memory aids and adherence with *p*-values being less than 0.05. Further research is needed to explore other potential factors influencing adherence. Model limitations should be addressed by a further investigation on why the model fails to classify non-adherence accurately. It might be useful to gather more data or refine the existing predictors.
